# Phosphoserine Aminotransferase Pathogenetic Variants in Serine Deficiency Disorders: A Functional Characterization

**DOI:** 10.3390/biom13081219

**Published:** 2023-08-04

**Authors:** Francesco Marchesani, Annalisa Michielon, Elisabetta Viale, Annalisa Bianchera, Davide Cavazzini, Loredano Pollegioni, Giulia Murtas, Andrea Mozzarelli, Stefano Bettati, Alessio Peracchi, Barbara Campanini, Stefano Bruno

**Affiliations:** 1 Department of Medicine and Surgery, University of Parma, 43124 Parma, Italy; 2 Department of Food and Drug, University of Parma, 43124 Parma, Italy; 3 Biopharmanet-TEC, University of Parma, 43124 Parma, Italy; 4 Department of Chemistry/Life Sciences and Environmental Sustainability, University of Parma, 43124 Parma, Italy; 5 The Protein Factory 2.0, Department of Biotechnology and Life Sciences, University of Insubria, 21100 Varese, Italy; 6 Institute of Biophysics, CNR, 56124 Pisa, Italy

**Keywords:** phosphorylated pathway, serine deficiency disorders, phosphoserine aminotransferase, Neu–Laxova syndrome, neurometabolic disorders

## Abstract

In humans, the phosphorylated pathway (PP) converts the glycolytic intermediate D-3-phosphoglycerate (3-PG) into L-serine through the enzymes 3-phosphoglycerate dehydrogenase, phosphoserine aminotransferase (PSAT) and phosphoserine phosphatase. From the pathogenic point of view, the PP in the brain is particularly relevant, as genetic defects of any of the three enzymes are associated with a group of neurometabolic disorders known as serine deficiency disorders (SDDs). We recombinantly expressed and characterized eight variants of PSAT associated with SDDs and two non-SDD associated variants. We show that the pathogenetic mechanisms in SDDs are extremely diverse, including low affinity of the cofactor pyridoxal 5′-phosphate and thermal instability for S179L and G79W PSAT, loss of activity of the holo form for R342W PSAT, aggregation for D100A PSAT, increased K_m_ for one of the substrates with invariant k_cat_s for S43R PSAT, and a combination of increased K_m_ and decreased k_cat_ for C245R PSAT. Finally, we show that the flux through the in vitro reconstructed PP at physiological concentrations of substrates and enzymes is extremely sensitive to alterations of the functional properties of PSAT variants, confirming PSAT dysfunctions as a cause of SSDs.

## 1. Introduction

**Figure 1:**
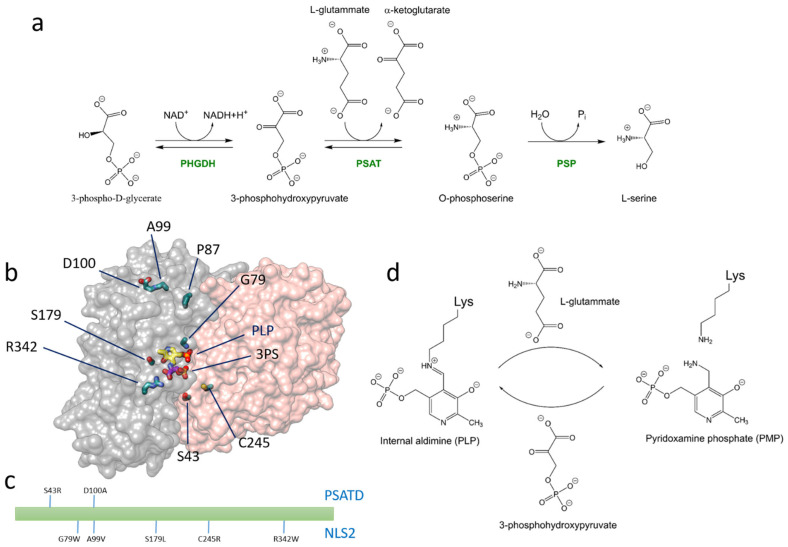
(**a**) Reactions of the PP. (**b**) Model of PSAT (pdb 8A5W) with PLP, 3-phosphoserine (3-PS) and the residues investigated in this work depicted as sticks. (**c**) Distribution of the substitutions along the PSAT sequence investigated in this work and associated with either PSATD (upper labels) or NLS2 (bottom labels). (**d**) Ligation states of PSAT investigated in this work, i.e., the internal aldimine form (PLP) and the pyridoxamine form (PMP).

The phosphorylated pathway (PP) converts the glycolytic intermediate D-3-phosphoglycerate into L-Ser through three sequential reactions catalyzed by the enzymes 3-phosphoglycerate dehydrogenase (PHGDH, EC 1.1.1.95), phosphoserine aminotransferase (PSAT, EC 2.6.1.52) and phosphoserine phosphatase (PSP, EC 3.1.3.3) (Figure 1a) [1]. Although the three enzymes are expressed in several tissues, the PP is particularly important in the central nervous system (CNS), consistently with the limited permeability of L-serine (L-Ser) through the blood–brain barrier [2], and the neurological phenotypes associated with genetic defects of either one of the three enzymes. We have recently shown that the three enzymes of the PP co-localize in cytoplasmic clusters yielding a metabolome we named ‘serinosome’ [3].

In the CNS, de novo synthesized L-Ser is the metabolic precursor of D-serine (D-Ser)—a co-agonist of the glutamatergic NMDA receptors [4]—that is produced by serine racemase (SR, EC 5.1.1.18) [5,6,7]. D-Ser is degraded by both SR via a β-elimination reaction [8] and D-amino acid oxidase (DAAO, EC 1.4.3.3) [9]. Altered D-Ser homeostasis in the CNS has been associated with several neurodegenerative and mental disorders—including schizophrenia [10,11], amyotrophic lateral sclerosis [12,13] and dementia [14], thus calling for a thorough investigation of the PP-SR-DAAO pathways to gain insight into the pathogenesis of these conditions. In addition to its role as a precursor of neurotransmitters, L-Ser is also the precursor of phosphoglycerides and sphingolipids, which are important membrane and myelin components and are also involved in cellular differentiation, proliferation, migration, and apoptosis ([1,15] and references herein).

Genetic defects of either PSAT, PHGDH, or PSP are associated with a group of neurometabolic disorders known as serine deficiency disorders (SDDs), hallmarked by a low concentration of L-Ser in the cerebrospinal fluid and plasma [16,17]. SDDs exhibit highly variable neurological phenotypes in the infantile forms, with retardation, microcephaly, and seizures. Progressive polyneuropathy manifests in adult patients [16,17]. Neu–Laxova syndrome (NLS) is the most severe expression within the spectrum of SDDs and includes a broad range of phenotypes such as lethality, severe neurological manifestations, seizures, and intellectual disability [18,19]. When NLS is associated with homozygous or compound heterozygous mutations of *PSAT1*, it is known as Neu-Laxova syndrome-2 (NLS2, OMIM # 616038). The early recognition of the milder forms of SDDs is crucial for their successful treatment with L-Ser repletion [20]. Since defects of all three enzymes of the PP can generate SDDs—producing overlapping phenotypes—the molecular understanding of the enzymes’ pathogenetic variants is relevant for the diagnosis, prognosis, and therapy of the affected individuals.

Although most occurrences of SDD are associated with pathogenic variants of PHGDH [16,17,21,22], a small but increasing number of cases were shown to involve mutations on the gene—*PSAT1*—encoding for PSAT, the homodimeric pyridoxal 5′-phosphate (PLP)-dependent enzyme that catalyzes the transamination of 3-phosphohydroxypyruvate (3-PHP) and L-glutamate (L-Glu) to 3-phosphoserine (3-PS) and α-ketoglutarate (Figure 1a). We have recently reported the characterization of the recombinant human ortholog, including the three-dimensional X-ray crystal structures of the substrate-free (pdb 8A5V) and 3-PS-bound forms (pdb 8A5W) [23]. SDDs associated with the genetic impairment of PSAT are known as PSAT deficiencies (PSATD, OMIM # 610992) and were first reported in two siblings heterozygous for a frameshift and a missense mutation of *PSAT1*, the latter encoding for the D100A variant [24]. One sibling exhibited microcephaly, psychomotor retardation, intractable seizures, and hypertonia. The younger sibling was promptly treated with glycine and serine, avoiding most of the manifestations and constituting the first evidence of treatment of PSATD [24]. The D100A PSAT variant was recombinantly expressed in *Escherichia coli*, and its V_max_ was 15% of that of the wild-type (wt) enzyme, with no difference in K_m_ for phosphoserine [24]. More recently, a case related to the S43R PSAT variant was classified as an intermediate between mild NLS and very severe PSATD [25,26], whereas the A15P variant was associated with a juvenile-onset form of SDD [27]. A patient carrying the compound heterozygous pathogenic variants T156M and A15P was diagnosed with PSATD and was reported to benefit from administration of L-Ser [20].

By studying a cohort of 12 unrelated families affected by NLS2, Acuna-Hidalgo and colleagues identified two missense *PSAT1* mutations, coding for variants A99L and S179L [28]. The Y70N and R342W variants were identified in Chinese families [29] and a screening of 15 families with NLS2 led to the identification of the G79W and C245R PSAT variants [26]. The same study confirmed the association of NLS2 with the A99V variant [26]. A yeast-based complementation assay on PSATD- and NLS2-associated PSAT variants provided a quantitative assessment of their activity [30]. The S179L variant was almost inactive, whereas the A99V exhibited 85% of the activity of wt PSAT [30].

In this work, we have recombinantly produced and thoroughly characterized selected variants reported in the literature as associated with either PSATD or NLS2, with the aim of identifying the specific loss-of-function mechanisms that might be responsible for SDDs pathogenesis. For comparison, we also characterized P87A PSAT, the only Uniprot-reviewed natural variant not associated with disease, and an alternatively spliced variant, PSAT alpha, lacking 46 amino acids between V290 and S337 and reported to be at least partially active [31].

## 2. Material and Methods

### 2.1. Proteins Expression and Purification

The pET28b-based expression vector for wt PSAT has been described elsewhere [32]. Those for PSAT variants were prepared by Genscript (Piscataway, NJ, USA) based on the same vector. The expression and purification steps have been already reported [23]. Briefly, the expression was carried out at 20 °C in *E. coli* BL21(DE3) tuner cells (Novagen™, Merck, Darmstadt, Germany) by addition of 0.2 mM isopropyl-β-D-1-thiogalactopyranoside (IPTG). The protein was then purified by IMAC (Talon superflow—Cytiva™) and dialyzed against 25 mM Tris pH 8, 300 mM NaCl, 1 mM TCEP and 4 µM PLP, concentrated, flash-frozen in liquid nitrogen and stored at −80 °C. To obtain the PLP and PMP forms, we added 3-PHP and L-Glu at 110 µM and 20 mM final concentration, respectively. The samples were dialyzed against 20 mM potassium phosphate buffer pH 7, then aliquoted and stored at −80 °C.

### 2.2. Dynamic Light Scattering

DLS measurements were collected with a Zetasizer Nano (Malvern Instruments™, Malvern, UK) coupled with a 633 nm laser, using NIBS detection (173° backscatter) at 25 °C. wt PSAT and its variants were centrifuged at 16,000× *g* for 45 min and then diluted at 23 µM concentration in a solution containing 50 mM NaH_2_PO_4_ and 300 mM NaCl, pH 7. Three independently prepared samples of each protein were analyzed, and, for each sample, three measurements were collected immediately after preparation and after 1 h.

### 2.3. Circular Dichroism (CD) Spectroscopy

Circular dichroism spectra were collected with a Jasco™ J-1500 spectropolarimeter equipped with a Peltier thermostatic unit set at 20 °C. The protein concentration was 5 µM in a buffered solution containing 20 mM potassium phosphate at pH 7. The spectra were collected between 250 and 180 nm. Each spectrum was the result of 3 averaged accumulations. Secondary structure estimation was performed by using the Dichroweb server (http://dichroweb.cryst.bbk.ac.uk, accessed on 12 September 2022), exploiting the CONTIN analysis program and the reference set optimized for 185–240 nm. All CD spectra were corrected for buffer background. For the estimation of the melting temperature, far-UV CD signal changes at 222 nm were monitored as a function of increasing temperature from 20 to 80 °C, with a ramp rate of 5 °C per minute.

For PSAT variants showing a two-step thermal unfolding the double Boltzmann equation (Equation (1)) was also applied to estimate the melting temperatures for the two transitions.

(1) $$\theta \;=\;\theta_{0}\;+\;A\;\left[\frac{f}{1\;+\;e^{\frac{T\;-\;T_{m1}}{k_{1}}}}\;+\;\frac{1\;-\;f}{1\;+\;e^{\frac{T\;-\;T_{m2}}{k_{2}}}}\right]$$

where *θ* is the ellipticity at 222 nm, *θ*_0_ is an offset, *A* is the amplitude, *f* is the fractional amplitude of the first phase, *T* is the temperature in °C, *Tm*_1_ and *Tm*_2_ are the melting temperatures of the two phases and *k*_1_ and *k*_2_ are the slopes of the two phases.

For variants exhibiting a monophasic behavior, the thermal denaturation curves were analyzed considering a two-state transition. Data were fitted to (Equation (2)).

(2) $$\theta =\theta_{0}+\frac{f}{1+e^{\frac{T-T_{m}}{k}}}$$

where *θ* is the ellipticity at 222 nm, *θ*_0_ is an offset, *f* is the amplitude of the thermal transition, *T* is the temperature in °C, *Tm* is the melting temperature and *k* is the slope of the phase. The analysis was performed using MATLAB software (version R2022a).

### 2.4. Absorption Spectra

UV-vis spectra of PSAT variants after purification were acquired in the 600–250 nm range using a Varian CARY 4000 spectrophotometer (Agilent™, Santa Clara, CA, USA) at 20.0 ± 0.5 °C. To evaluate PLP uptake, 1 µM or 5 µM PLP was added in selected experiments and allowed to equilibrate for 2 min before collection of the spectrum. The absorption spectra of PLP forms of PSAT variants were collected in a solution containing 50 mM MES, 50 mM HEPES, 50 mM Bicine, 100 mM NaCl, at pH 5.9. The saturation fraction with PLP was assessed through the ratio between absorbance at 408 nm and 280 nm, considering wt PSAT as 1.

### 2.5. Activity Assays for the Determination of Kinetic Parameters with L-Glu

As indicated in our previous work [23], PSAT follows a ping-pong kinetic mechanism with substrate inhibition; the calculation of intrinsic K_m_s, k_cat_s and K_i_s requires the global fitting of multiple datasets of initial rate on one substrate concentration at different concentrations of the second substrate. This analysis being beyond the scope of the present work, we calculated the apparent kinetic parameters as detailed below, with the awareness that substrate inhibition is also in play with the variants but is likely to be a minor component of the total effect of substitutions on the enzyme functional properties in vivo. For the wt enzyme and catalytically active variants (i.e., S43R, P87A, A99V, D100A, C245R PSAT), the apparent kinetic parameters (K_m_, k_cat_ and catalytic efficiency) towards L-glutamate were determined using a continuous assay by coupling the production of α-ketoglutarate with the reaction of glutamate dehydrogenase (GDH type II from bovine liver—MERCK™; one unit per assay), as already described [23]. Briefly, the enzyme assays were performed at 37 °C in a reaction mixture containing 50 mM HEPES pH 7, 100 mM KCl, 1 mM DTT, 32 mM NH_4_Cl, 0.1 mM NADH, 0.17 mM PLP, 0.11 mM 3-PHP and L-Glu, ranging from 0.4 to 80 mM. Assays were performed using a Cary 4000 spectrophotometer (Agilent™, Santa Clara, CA, USA). After 3 min of preincubation time in the cuvette holder, the reactions were started by adding 80 nM PSAT for all PSAT variants, except for the poorly active C245R PSAT, for which the concentration was increased to 320 nM. The initial rates were estimated using an extinction coefficient for NADH of 6220 M^−1^cm^−1^ at 340 nm, after subtracting the rate of the pre-incubation phase. Measurements were performed using a Cary 4000 spectrophotometer (Agilent™, Santa Clara, CA, USA).

The initial rates as a function of L-Glu concentrations were then fitted to the Michaelis–Menten equation (Equation (3)), to estimate the apparent kinetic parameters. In the equation, *v*_0_ is the initial rate, *K_m_* is the Michaelis–Menten constant, *V_max_* is the reaction rate at substrate saturation and [L-Glu] is the concentration of L-glutamate.

(3) $$v0\;=\;\frac{Vmax\;\left[L-Glu\right]}{K_{m}\;+\;\left[L-Glu\right]}$$

To obtain the values of *k_cat_*, the maximal velocities (*V_max_*) expressed in μM/s were divided by the enzyme concentrations (in μM). The catalytic efficiencies were obtained by dividing the values of *k_cat_* (s^−1^) for the values of *K_m_* (in molarity). Data were fitted using SigmaPlot software.

### 2.6. Single Kinetic Fitting for the Evaluation of Kinetic Parameters with 3-PHP

To obtain the apparent kinetics parameter for the substrate 3-PHP, a single kinetic fitting model was implemented using the online tool PCAT (https://www.weizmann.ac.il/Biomolecular_Sciences/Schreiber/content/pcat, accessed on 12 November 2022), which allows the resolution of the Schnell—Mendoza equation (Equation (4)) using a numerical or analytical method [33]. In our case, the analytical or numerical solutions resulted in almost identical values of kinetic parameters. In this work, we considered the analytical solution:

(4) $$\left[S\right]\left(t\right)=K_{m\;}W\left(\frac{S_{0}}{K_{m}}\;e^{\left(\frac{S_{0}\;-\;V_{max}t\;}{K_{m}}\right)}\right)$$

where *W* is the omega function, *K_m_* is the Michaelis–Menten constant, *S*_0_ is the initial concentration of substrate, *V_max_* is the rate at saturating substrates concentrations and *t* is the time in seconds.

The kinetic assays were carried out in the same buffer solution as described above, with minimal changes, i.e., the presence of 0.2 mM NADH instead of 0.1 mM, the concentration of 3-PHP ranging from 60 to 80 µM and the concentration of L-Glu which was equal to 5-fold the K_m L-Glu_ value, in order to almost saturate the enzyme variants (10 mM for wt enzyme; 14 mM for A99V and P87A PSAT; 65 mM for S43R PSAT and 108 mM for C245R PSAT). Briefly, all the assay components except for PSAT were preincubated at 37 °C for 3 min and the reaction was initiated by the addition of 80 nM PSAT. The rates were measured from the slopes of the initial linear portion of each curve as described above using the extinction coefficient for NADH (6220 M^−1^cm^−1^).

### 2.7. In Vitro Reconstruction of the Phosphorylated Pathway

Recombinant human PHGDH and PSP were produced as reported elsewhere [34,35]. To reconstruct the PP kinetic profile under physiological conditions, a continuous assay was performed following the reaction of the rate-limiting enzyme PHGDH in the presence of PSAT (wt or variants) and PSP. The assay was performed by using astrocytic intracellular concentrations of the three enzymes of the PP, i.e., 0.82 µM PHGDH, 1.14 µM PSAT, and 0.12 µM PSP [36]. The buffered solution also contained 50 mM HEPES, 100 mM KCl, 1 mM DTT, 0.3 mM NAD^+^, 2 mM L-Glu, 0.1 mg/mL BSA, 0.3 mM MgCl_2_, pH 7. The assay reagents were preincubated at 37 °C for 3 min and the reaction was initiated by adding 0.54 mM D-3-phosphoglycerate (3-PG). The reaction was followed for 15 min by measuring absorption at 340 nm: this value was converted to NADH concentration using an extinction coefficient of 6220 M^−1^cm^−1^.

### 2.8. Size Exclusion Chromatography (SEC)

SEC analyses were performed on an Akta Pure 25M chromatographic system (GE Health Sciences™, Chicago, IL, USA) equipped with a Superdex 200 Increase 5/150 GL column (GE Health Sciences™) with a mobile phase consisting of 50 mM HEPES, pH 7 and 300 mM KCl, at a flow rate of 0.3 mL/min. The separation was conducted at room temperature and the column effluent was monitored at 280 nm. Proteins were loaded at 23 µM, resulting in an elution concentration of around 7.5 µM. The calibration curve consisted of thyroglobulin, aldolase, bovine serum albumin, alcohol dehydrogenase, trypsinogen, ovalbumin.

## 3. Results

### 3.1. Variant Selection and Predicted Functional Impact of Substitutions

The PSAT variants reported in Table 1 and Figure 1 were selected based on reported cases of NLS2 and PSATD. The functional impact based on the evolutionary conservation of the affected residues was predicted by the Mutation Assessor and the PolyPhen-2 algorithms [37,38] (Table 1). Published predictions on the associated structural perturbations based on the analysis of the pdb model 3E77 [26] are also reported in Table 1.

### 3.2. Protein Expression and Purification

PSAT has been reported in two splice variants, PSAT beta and PSAT alpha, the latter lacking 46 amino acids between V290 and S337 [31]. We performed several attempts at purifying recombinant PSAT alpha, but the protein was always found in the non-soluble fraction. Neither the use of different *E. coli* strains nor alternative purification strategies (i.e., recovery from inclusion bodies) were successful. We therefore concluded that this variant is not soluble and not functional, at least in its recombinant form. Therefore, we focused on the variants of PSAT beta, assuming it to be the functional form.

All variants were expressed and purified with similar yields as wt PSAT (around 80 mg per liter of culture) and a purity ranging from 90% to 97%, except for variant S179L, consistently produced at 5-fold lower yields in comparison to wt PSAT and at a lower purity (Figure S1, Supplementary Materials). By using our expression system, recombinant D100A PSAT was expressed at similar levels as wt PSAT, differently from a previous study that suggested significant instability [24]. D100A PSAT exhibited a greater electrophoretic mobility in comparison to wt PSAT and all other variants (Figure S1, Supplementary Materials). Mass spectrometry experiments confirmed its mass and allowed us to attribute the anomaly to the substitution of a negatively charged side chain with a hydrophobic one, similar to what has been observed for other proteins [39].

### 3.3. PSAT Spectroscopy

The UV-vis absorption spectra of purified PSAT variants varied significantly, with different relative contributions of the bands at 408 nm and 339 nm, suggesting a different relative amount of PLP and PMP [23] (Figure 1d and Figure S2, Supplementary Materials). The spectra of variants S179L and G79W PSAT exhibited very low intensity in the 340–500 nm range in comparison to absorption at 280 nm, indicating low PLP saturation.

**Figure 2:**
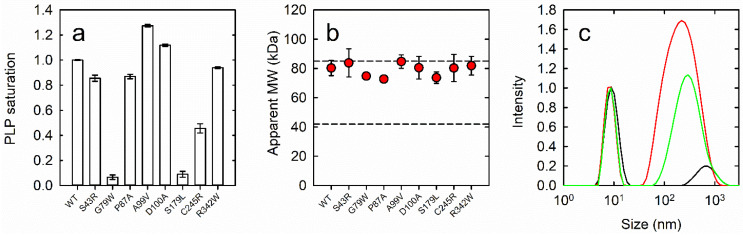
(**a**) PLP saturation for all PSAT variants (normalized to that of wt PSAT) in a solution containing 50 mM MES, 50 mM HEPES, 50 mM Bicine and 100 mM NaCl, pH 5.9. (**b**) Apparent molecular weight of PSAT variants loaded in a Superdex 200 increase column in a buffer solution containing 50 mM HEPES, 300 mM KCl, pH 7. The dashed lines indicate the theoretical molecular mass of the dimer (86 kDa) and the monomer (43 kDa). (**c**) DLS signal analysis of 23.3 µM PSAT variants in a buffer containing 50 mM NaH_2_PO_4_, 300 mM NaCl, pH 7. The DLS intensity of wt (black), D100A (red) and S179L (green) collected after 1 h. Data were normalized to the peak intensity of the particle corresponding to dimeric PSAT.

To compare the PLP saturation of the variants, the pure internal aldimine forms and the PMP forms were produced by the addition of either 0.11 mM 3-PHP or 20 mM L-Glu to aliquots of each protein solution, respectively (Figure 1d) [23]. The ratio of absorption intensity at 280 nm and 408 nm for the PLP forms was used to assess PLP saturation (Figure 2a). Variants C245R, S179L, and G79W PSAT were 50%, 10%, and 5% saturated compared to wt PSAT, respectively. Variants A99V and D100A PSAT exhibited a higher A_408 nm_/A_280 nm_ ratio than wt PSAT, indicating either a higher saturation or a slightly different extinction coefficient of the PLP moiety at 408 nm. For all PSAT variants, the saturation did not increase by incubation with free PLP up to 5 μM concentration for 1 h (Figure S3, Supplementary Materials).

### 3.4. Secondary Structure

The assessment of the secondary structure of all PSAT variants was carried out by recording circular dichroism (CD) spectra in the 185–250 nm range of both the PLP and PMP forms. The percentages of secondary structure elements resulting from the mean of three independently prepared samples are reported in Table S1 for each variant in the PLP-form and in Table S2 for the PMP-form. In general, no significant changes in secondary structure neither for the PLP nor for the PMP forms was observed in comparison to wt PSAT. The S179L PSAT variant was the only one exhibiting a change, albeit marginal, in secondary structure (Table S1). The PMP forms also were invariant in secondary structure.

### 3.5. Oligomeric State

Since it was predicted that some variants might be prone to dimer dissociation (Table 1 and [26]), we performed size exclusion chromatography (SEC) experiments (Figure 2b and Figure S4, Supplementary Materials). Proteins were loaded onto the column at 23 µM, resulting in an elution concentration of around 7.5 µM. Based on a calibration curve in the 14–140 kDa range (Figure S4, upper panel), the elution time of wt PSAT (around 6 min) corresponded to a molecular mass of 80 ± 5 kDa, consistent with a dimer (86 kDa). All variants eluted roughly at the same elution time as wt PSAT, suggesting that they all retain a dimeric oligomerization state in the low µM concentration range (Table 2, Figure 2b and Figure S4, Supplementary Materials).

Since D100A PSAT exposes a hydrophobic residue toward the bulk solution (Figure 1), and structural perturbations produced by substitutions in other variants might also indirectly result in altered surface hydrophobicity, we assessed protein aggregation by dynamic light scattering (DLS) experiments (Table 2). Intensity particle size distributions (Figure S5a) for all variants revealed the presence of a population with a hydrodynamic diameter of around 7.45 nm, which corresponds to 74 kDa. These values, albeit slightly variable among variants (Table 2), were all consistent with a PSAT dimer, which has a calculated mass of 86 kDa. Populations with a higher hydrodynamic diameter ranging from 0.4 to 1 µm—corresponding to aggregates of around 500–80,000 copies of PSAT dimers—were also observed for wt and variants of PSAT. These populations accounted for a small share of the total signal intensity, except for D100A PSAT, which exhibited a population of 258.4 nm in size (around 3500 dimers) accounting for around 74% of the overall signal intensity. After 1 h (Figure S5b), no major changes were observed, except for S179L PSAT, which exhibited a tendency to aggregate over time. This behavior may correlate with its perturbed secondary structure and the lower expression yields.

### 3.6. Thermal Stability

**Figure 3:**
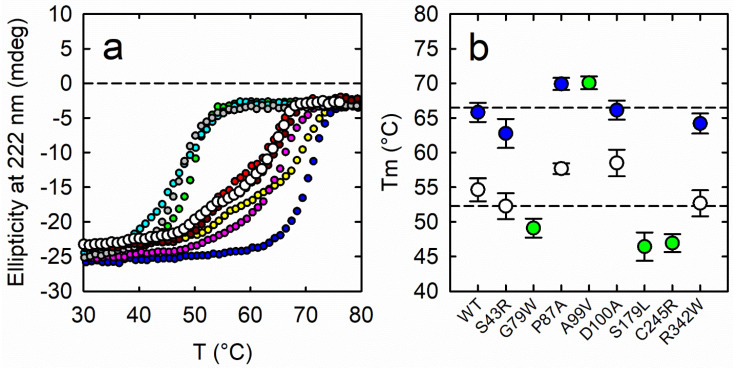
(**a**) Temperature ramps in the 30–80 °C range of PSAT wt (black), S43R (red), G79W (green), P87A (yellow), A99V (blue), D100A (pink), S179L (light blue), C245R (grey), R342W (dark red) in the PLP-bound form followed as CD signal intensity at 220 nm. (**b**) Calculated melting temperatures of PSAT variants. The green circles represent the single T_m_ value obtained for variants exhibiting a monophasic behavior. For the variants exhibiting a biphasic behavior, the lower (white circles) and higher (blue circles) T_m_s are reported.

Reduced thermodynamic stability of PSAT pathological variants was proposed as a pathogenetic mechanism for SDDs (Table 1 and [26]). Therefore, we performed thermal denaturation experiments by monitoring circular dichroism at 222 nm, a wavelength diagnostic of changes in secondary structure in PSAT (Figure 3). Experiments were carried out for the PLP-bound form (Figure 3a) although the poorly PLP-saturated variants (G79W and S179L) should be essentially regarded as apo-forms. As already observed for wt PSAT [23], the denaturation curves of most variants exhibited a biphasic behavior, which we associated with partial denaturation followed by monomerization, in turn preceding full unfolding, as already observed for other PLP-dependent enzymes [40] and for PSAT from *Entamoeba histolytica* [41]. In the case of wt PSAT, the protein secondary structure is stabilized, with respect to the second step, by PLP binding [41], with T_m_ increasing significantly in comparison to non PLP-bound variants. The traces of PSAT variants were fitted using a double Boltzmann equation (Equation (1)) except for A99V, G79W, S179L, and C245R, which exhibited a monophasic behavior (Figure S6) and where therefore fitted with a single Boltzmann equation (Equation (2)). Variants G79W, S179L and C245R showed a very low saturation with PLP (Figure 1a) and, indeed, the only denaturation phase roughly coincides with the first denaturation phase of the wt PSAT (around 52 °C). Accordingly, for PSAT variants exhibiting a biphasic behavior, the denaturation step with T_m_ around 66 °C is likely stabilized by PLP. This transition was almost unchanged among PLP-bound variants, indicating that amino acid substitutions per se do not significantly impact protein stability (Figure 3b). A notable case was A99V, whose denaturation curve was monophasic, with the only detectable T_m_ occurring at the highest temperature for the other variants. We speculate that this might indicate a stronger interaction with PLP [23].

### 3.7. Enzyme Activity

**Figure 4:**
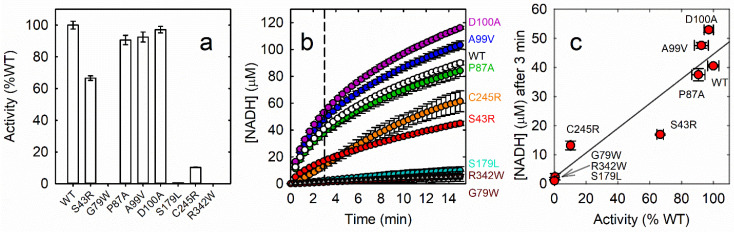
(**a**) Enzyme activity of 80.8 nM PSAT variants in a solution containing 50 mM HEPES, 100 mM KCl, 100 µM 3-PHP, 130 µM NADH, 170 µM PLP, 20 mM L-Glu, 32 mM NH4Cl and 1.5 mM DTT, pH 7. (**b**) In vitro reconstruction of the phosphorylated pathway mimicking physiological conditions following the reaction of the rate limiting enzyme PHGDH in the presence of PSAT (wt or variants) and PSP. The reactions were performed in 50 mM HEPES, 100 mM KCl, 0.3 mM MgCl2, 0.1 mg/mL BSA, 0.54 mM 3-PG, 0.3 mM NAD and 2 mM L-Glu, pH 7 at 37 °C. Reactions were started adding 3-PG. The concentrations of PHGDH, PSAT and PSP were 0.82 µM, 1.14 µM and 0.12 µM, respectively. (**c**) Correlation between the activity of PSAT variants and NADH production by the PP after 3 min.

To preliminarily assess the activity of the PSAT variants, enzyme assays were carried out at fixed concentrations of substrates, i.e., 0.1 mM 3-PHP and 20 mM L-Glu [23], that correspond to saturating concentrations for the wt enzyme (Figure 4a). Under these conditions, variants S179L, G79W and R342W were virtually inactive. S43R and C245R PSAT were 60% and 10% as active as wt PSAT. All other variants did not exhibit any significant difference in activity in comparison to wt PSAT. To estimate if low PLP saturation is due to a reduced affinity, the same activity assays were carried out in the absence of added PLP and in the presence of 500 µM PLP after 1 h incubation. No significant increase in activity was observed (Figure S7, Supplementary Materials), indicating that either the active site is not accessible to PLP after protein folding or—for the non-saturated forms—the dissociation constant is significantly higher than for wt PSAT. A 50% decrease in activity was observed for the S43R variant in the presence of 500 μM PLP and a lower decrease was similarly observed for A99V (Figure S7, Supplementary Materials). Although the mechanism of this inhibition is not known, free PLP concentration in cells is in the low micromolar range.

For the active variants, a complete kinetic characterization was performed, determining K_m_ and k_cat_ values for the two substrates 3-PHP and L-Glu (Table 3, Figures S8 and S9, Supplementary Materials). Most of the active variants did not exhibit any perturbation of kinetic parameters, except for S43R and C245R. S43R exhibited a 3-fold higher K_m_ for 3-PHP and a 5-fold higher K_m_ for L-Glu. This finding is consistent with the observation that the substitution likely perturbs the active site, affecting the binding of both substrates. C245R PSAT, on the other hand, exhibited a 10-fold higher K_m_ for L-Glu, suggesting that replacement of the small Cys sidechain with the much bulkier Arg in the vicinity of the active site may differentially affect the binding of two substrates. Only in the C245R variant the increase in K_m_ is associated with a 2.7- and 4.7-fold decrease in k_cat_ for PHP and L-Glu, respectively, also hampered by a 50% reduced cofactor saturation. As a result, considering the catalytic efficiency (k_cat_/K_m_) this variant is 40-fold less efficient than the wt PSAT.

### 3.8. In Vitro Reconstruction of the Phosphorylated Pathway

To assess the effect of the different PSAT variants on L-Ser synthesis, we performed a continuous assay including the three enzymes—PHGDH, PSAT, and PSP—in the presence of the substrates 3-PG, NAD^+^ and L-Glu. The reaction was followed by monitoring NADH production at 340 nm by PHGDH, as previously described [3]. We have already shown that this assay affords similar rates as the one that monitors the formation of the final product phosphate since NADH accumulation is dependent on the following enzymatic steps [3]. The enzyme concentrations we used were those measured in differentiated astrocytes (i.e., 0.82 µM PHGDH, 1.14 µM PSAT and 0.12 µM PSP) [36]. The substrates concentrations were also physiological, with the exception of 3-PG, at 0.54 mM, the upper limit of physiological range [42]. Each assay was performed using different PSAT variants at the same concentration (Figure 4b). Since we could not observe a steady-state phase during PP kinetics under these conditions, the NADH production after the fixed time of 3 min was recorded. The S179L, R342W and G79W PSAT variants did not allow the PP to proceed at a significant rate. The C245R and S43R ones slowed down the PP by about 60%, consistently with their altered kinetic parameters in comparison to wt PSAT (Table 3). The P87A PSAT led to the production of NADH at a similar rate as wt PSAT. Finally, D100A and A99V PSAT produced NADH at a slightly higher rate than wt PSAT. Notably, NADH production by the PP after 3 min correlates well (r^2^ = 0.89) with the activity of PSAT variants (Figure 4c), confirming that the pathway is extremely sensitive to poorly active forms whose reduced activity is thus responsible for an overall decrease in L-Ser concentration.

## 4. Discussion

In this work we characterized the structural and functional properties of recombinant PSAT variants associated with SDDs in search for clues of their in vivo pathogenetic mechanisms. For comparison, the P87A natural variant, reported in UniProt as non-pathogenic, was also investigated, resulting in no significant difference with respect to wt PSAT in terms of stability, dimerization, PLP saturation and enzyme activity. For all the other variants, the putative pathogenetic mechanisms inferred in this work are reported in Table 1.

**Figure 5:**
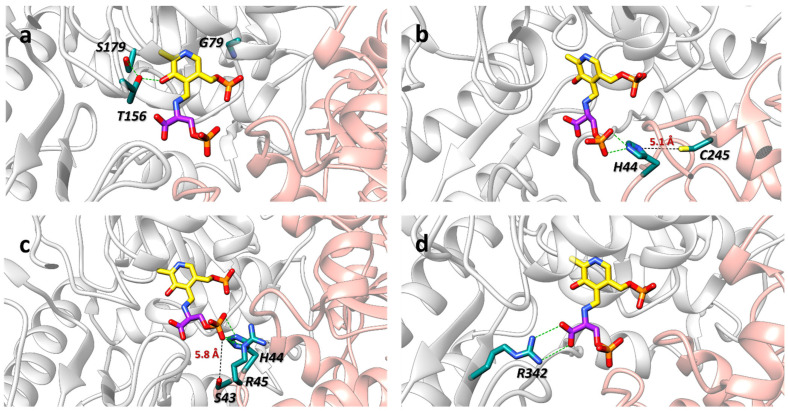
Close-up of a selection of residues analyzed in this work: Ser179 and Gly79 (**a**), Cys245 (**b**), Ser43 (**c**) and Arg342 (**d**). All residues are involved in variants that result in less active (or inactive) enzymes. The two protomers of PSAT are shown in transparency in grey and red; the PLP covalently bound to 3-PS as external aldimine is shown in yellow, while 3-PS is shown in magenta. Distances are highlighted as discontinuous black lines, while H-bonds are highlighted as discontinuous green lines (pdb 8A5W).

The S179L and G79W variants—both associated with NLS2 [28,30]—were purified as the apo form and could not be saturated by the addition of free PLP. They were enzymatically inactive and prevented the reconstructed PP from producing NADH. Additionally, S179L PSAT exhibited a tendency to aggregate over time, a property not shared by G79W PSAT. For both variants, the structural basis of the loss of function appears straightforward. S179 PSAT forms a hydrogen bond with T156, which is directly involved in a H-bond with the O3 of PLP [23]: thus, the substitution with a hydrophobic residue likely results in the loss of the interaction between PSAT and PLP (Figure 5a). G79 is located in the phosphate binding site of the cofactor PLP, and the substitution with a bulkier tryptophan residue is likely to lead to steric hindrance, hampering PLP binding (Figure 5a). Poor PLP binding was correctly predicted for G79W PSAT [26].

C245R PSAT—reported as associated with NLS2 [26]—was only 40% saturated with PLP and full saturation could not be achieved by incubation with a large excess of free PLP. At variance with computational predictions [26], C245R PSAT did not exhibit dimer instability, neither in DLS experiments nor in SEC experiments. Its most notable alteration is a 40-fold reduction in catalytic efficiency, contributed by both an increase in K_m_ for L-Glu and a 5-fold decrease in k_cat_. The partial saturation with PLP only accounts for part of the effect on k_cat_, thus the substitution also directly affects substrate binding and the turnover. C245 is located approximately at the interdimeric interface, 5.1 Å away from H44, which is relevant for the stabilization of the enzyme-substrate complex via an ionic interaction with the phosphate group of 3-PS (Figure 5b). It is possible that the substitution with a positively charged residue results in an electrostatic repulsion with H44 that does not allow for the stabilization of PSAT with its substrates, thus explaining the higher K_m_ for L-Glu. It should be pointed out that the physiological concentration of L-Glu in astrocytes is around 2 mM [3], and the overall activity of the PP in vivo is therefore expected to be impaired by the C245R PSAT variant. Consistently, the metabolic flux in the reconstructed PP was 50% reduced when wt PSAT was substituted with C245R PSAT.

The S43R PSAT—associated with PSATD [26,30]—was 80% PLP-bound in comparison with wt PSAT. It exhibited a similar thermal stability and no apparent perturbation of the secondary structure with respect to the wt enzyme and, contrary to computational predictions [26], dimer destabilization was not observed. However, a 3- and 5-fold increase in K_m_ was observed for PHP and L-Glu, respectively, with unaltered k_cat_s. The difference in K_m_ significantly affected the metabolic flux of the reconstructed PP, yielding a 50% decrease in NADH production at pseudo-physiological concentrations of substrates. S43 is located 5.8 Å from the PLP moiety and is not involved in direct interactions with either the PLP or the substrates (Figure 5c). However, a bulkier and positively charged side chain such as Arg might extend up to the active site, thus producing steric hindrance and electrostatic repulsion to the 3-PS phosphate binding site residues (H44, R45). Notably, the S43R variant, identified in a serious form of PSATD, and the C245R variant identified in NLS2, show a comparable behavior in the reconstructed pathway, despite a more pronounced effect of C245R on the catalytic efficiency parameter. This result suggests that differences in K_m_, more than in k_cat_ values, are responsible for the detrimental effects on the rate of the flux through the pathway.

The R342W variant, associated with NLS2, was around 80% saturated by PLP in comparison to wt PSAT, but its activity and the corresponding flux through the reconstructed PP were negligible. R342 is involved in a two-point electrostatic interaction with the carboxylate group of the substrate (Figure 5d). The loss of the positively charged side chain and the steric hindrance brought about by the indolic moiety likely justify the lack of activity.

The D100A variant was reported in two siblings heterozygous for this mutation and for a frameshift mutation of *PSAT1* [24]. It had been produced in recombinant form in a previous study, where it was found to exhibit a V_max_ 15% of that of wt PSAT [24]. However, our recombinant enzyme exhibited similar kinetic parameters as wt PSAT. Consistently, the rate of the reconstructed PP was similar to that in the presence of wt PSAT. D100A PSAT was the only variant aggregating significantly more than wt PSAT in DLS experiments, possibly due to the substitution of a charged side chain with a hydrophobic one at the protein surface. Although we could not detect any other functional difference with wt PSAT, it is possible that the tendency to aggregate might be responsible for the observed phenotypes in vivo, particularly in consideration of the formation of the cluster observed for the PP enzymes [3]. It should be noted that both siblings were compound heterozygotes for the variants, that were thus inherited in an autosomal recessive way from the parents. Notably, none of the parents were reported to suffer from symptoms related to serine deficiency. Thus, apparently, the tendency to aggregate of the D100A variant is symptomatic only when the other allele codes for a completely non-functional variant. Further studies co-expressing the wt and the D100A variant might help to shed light on this condition.

The A99V PSAT exhibited unexpected features, considering the instability predicted by computational tools [26]. The individuals expressing this variant are affected by NLS forms milder than, e.g., the one associated with the S179L substitution. Nevertheless, they died soon after birth or within some weeks. The recombinant protein characterized in this work not only was marginally more active than wt PSAT, but it also exhibited a significantly higher T_m_ (70 vs. 63 °C). In addition, the denaturation curves exhibited a monophasic behavior, possibly indicative of higher affinity for PLP or altered folding/unfolding pathways in comparison with wt PSAT. Unlike D100A, no tendency for aggregation was observed. The association of the A99V substitution with SDDs might suggest that this position—adjacent to D100—is a hotspot for protein–protein interaction, either within the ‘serinosome’ [3]—albeit the reconstructed PP showing no significant differences in comparison with wt PSAT—or with other protein regulators that have not yet been identified. Alternatively, the increased stability of A99V PSAT might alter as-yet-unknown regulation or degradation pathways at the cellular level. Further in-cell studies will be needed to clarify the pathogenetic mechanism associated with this variant.

In conclusion, the association of PSAT variants with SDDs appears to stem from different possible pathogenetic mechanisms, including a low PLP affinity and consequent thermal instability (S179L and G79W PSAT), a loss in activity of the holo form (R342W PSAT), aggregation (D100A PSAT), impaired kinetic parameters (S43R PSAT, C245R PSAT) and increased rigidity/higher thermal stability (A99V). This complex behavior calls for future cellular in-depth studies of the PSAT variants and their assessment within the PP to quantitatively evaluate their overall effect on L-Ser production. It should also be pointed out that most predictions based on computational tools and structure analysis (Table 1) did not correctly predict the functional impairment. We cannot rule out that variants with functional properties close to wt PSAT (D100A and A99V) might exhibit an altered interactome in vivo, particularly in light of the recent finding that the PP enzymes form clusters, possibly with the involvement of yet-unidentified proteins [3]. Finally, the results on the reconstruction of the PP confirmed that it is sensitive to PSAT activity, with less active variants affecting the pathway at physiological concentrations of enzymes and substrates. This is the first report addressing the different origin of catalytic inactivity of PSAT variants, which also show an altered PLP affinity for the enzyme.

It is worth mentioning that the administration of PLP precursors (in addition to L-Ser) might help in the management of certain types of PSAT-associated SDDs. Supplementation with pyridoxine has already been successfully applied to the treatment of disorders caused by specific pathogenetic variants of PLP-dependent enzymes—for example, in a subset of patients with primary hyperoxaluria type I [43]. Such treatment is likely to have beneficial outcomes because of the dual effect of PLP binding on both folding and catalytic activity of the defective enzyme.

## Figures and Tables

**Table 1:** Human PSAT variants, associated pathologies and predicted enzyme alterations.

			Functional Impact Predictions			
Variant	Pathology	Localization	Mutation Assessor [37]	PolyPhen-2 [38]	Analysis of pdb 3E77 [26]	Pathogenetic Mechanism Elucidated in This Work
S43R	PSATD [26,30]	PLP domain	High	Possibly damaging	Dimer instability	Altered kinetic parameters
G79W	NLS2 [26,30]	PLP domain	High	Probably damaging	Reduced PLP binding	Poor PLP binding, catalytical inactivity, thermal instability
P87A	Not associated with pathology (Uniprot)	PLP domain	Medium	Probably damaging	-	-
A99V	NLS2 [26,28]	PLP domain	Medium	Probably damaging	Protein instability	Altered interactome?
D100A	PSATD [24,30]	PLP domain	Low	Probably damaging	-	Aggregation/altered interactome?
S179L	NLS2 [28,30]	PLP domain	High	Probably damaging	-	Poor PLP binding, catalytical inactivity, thermal instability, aggregation
C245R	NLS2 [26]	PLP domain	Medium	Probably damaging	Dimer instability	Incomplete PLP binding, thermal instability, altered kinetic parameters
R342W	NLS2 [29]	C-terminus	High	Probably damaging	-	Catalytical inactivity

**Table 2:** Apparent oligomeric state of human PSAT variants (and their aggregates) by SEC and DLS.

Variant	SEC	DLS		
	Est. MW (kDa ±S.E.M.)	Est. MW (kDa ± S.E.M.)	Aggregation (% Signal Intensity)	
			t_0_	1 h
WT	80 ± 5	73 ± 1	21	25
S43R	84 ± 10	76 ± 4	25	26
G79W *	75 ± 1	63 ± 0	30	41
P87A	73 ± 0	78 ± 5	19	23
A99V	85 ± 5	76 ± 2	19	24
D100A	81 ± 8	66 ± 1	74	78
S179L *	74 ± 4	97 ± 0	28	70
C245R	80 ± 9	62 ± 5	15	26
R342W	82 ± 6	72 ± 1	34	37

* these variants should be considered as apo forms.

**Table 3:** Kinetic constants for the two substrates 3-PHP and L-Glu for the forward reaction catalyzed by human PSAT variants at 37 °C in a solution containing 50 mM HEPES, 100 mM KCl, 1 mM DTT, 0.17 mM PLP, pH 7.0. Parameters that differ significantly (≥3-fold) from the wt enzyme are highlighted in red.

	K_m_		k_cat_ (s^−1^)		k_cat_/K_m_ (M^−1^ s^−1^)	
	3-PHP (µM)	L-Glu (mM)	3-PHP	L-Glu	3-PHP	L-Glu
WT	9.9 ± 0.5	2.4 ± 0.3	29.5 ± 0.5	19.1 ± 0.5	3.0 ± 0.2 × 10^6^	8.0 ± 1.0 × 10^3^
S43R	31.0 ± 4.0	12.0 ± 2.0	27.0 ± 2.0	20.0 ± 1.0	0.9 ± 0.1 × 10^6^	1.6 ± 0.3 × 10^3^
P87A	8.5 ± 0.2	2.8 ± 0.4	25.5 ± 0.6	21.9 ± 0.1	3.0 ± 0.0 × 10^6^	8.0 ± 1.0 × 10^3^
A99V	14.8 ± 0.8	2.8 ± 0.4	34.0 ± 1.0	24.0 ± 1.0	2.3 ± 0.0 × 10^6^	9.0 ± 1.0 × 10^3^
D100A	10.5 ± 0.6	3.3 ± 0.3	23.0 ± 1.0	20.6 ± 0.5	2.2 ± 0.0 × 10^6^	6.2 ± 0.6 × 10^3^
C245R	13.0 ± 4.0	22.0 ± 5.0	11.0 ± 3.0	4.1 ± 0.4	0.9 ± 0.1 × 10^6^	0.2 ± 0.1 × 10^3^

## Data Availability

Data is contained within the article or Supplementary Materials.

## Supplementary Materials

The following supporting information can be downloaded at: https://www.mdpi.com/article/10.3390/biom13081219/s1, Figures S1–S9, Table S1: Secondary structure analysis of 5 µM PSAT-PLP CD spectra, and Table S2: Secondary structure analysis of 5 µM PSAT-PMP CD spectra.

## Abbreviations

CD, circular dichroism; CNS, central nervous system; DAAO, D-amino acid oxidase; DLS, dynamic light scattering; D-Ser, D-serine; L-Glu, L-glutamate; MW, molecular weight; NAD+\NADH, nicotinamide adenine dinucleotide; NLS, Neu–Laxova syndrome; NLS2, Neu–Laxova syndrome-2; 3-PS, 3-phosphoserine; 3-PG, 3-phosphoglycerate; PHGDH, 3-phosphoglycerate dehydrogenase; 3-PHP, 3-phosphohydroxypyruvate; PLP, pyridoxal-5′-phosphate; PP, phosphorylated pathway; PSAT, phosphoserine aminotransferase; PSATD phosphoserine aminotransferase deficiency; PSP, phosphoserine phosphatase; SDD, serine deficiency disorders; SEC, size exclusion chromatography; L-Ser, L-serine; SR, serine racemase; WT, wild type.
