# Supplementary material for: Phosphoserine Aminotransferase Pathogenetic Variants in Serine Deficiency Disorders: A Functional Characterization
Source: Biomolecules. 2023 Aug 4;13(8):1219. doi: 10.3390/biom13081219 (PMC10452355; doi:10.3390/biom13081219)
Supplement: Supplementary file 1 [file biomolecules-13-01219-s001.zip › biomolecules-2495937-supplementary.pdf]

Supplementary Materials

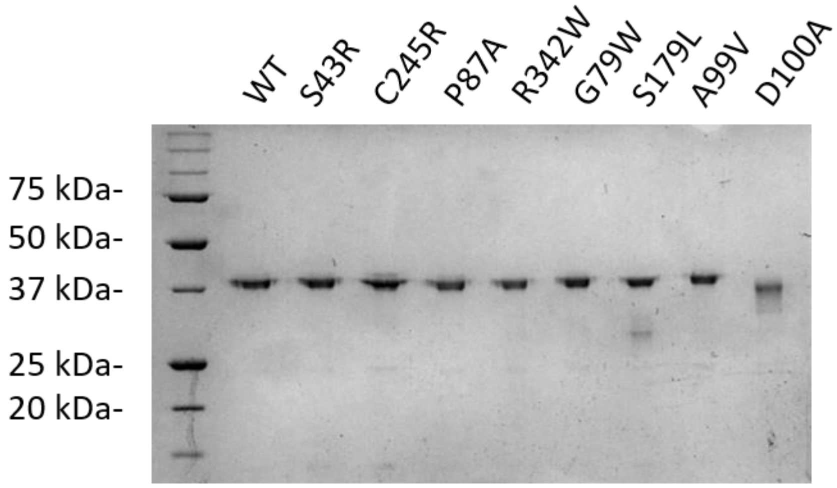

Figure S1: SDS-PAGE analysis of 150 ng of purified PSAT variants.

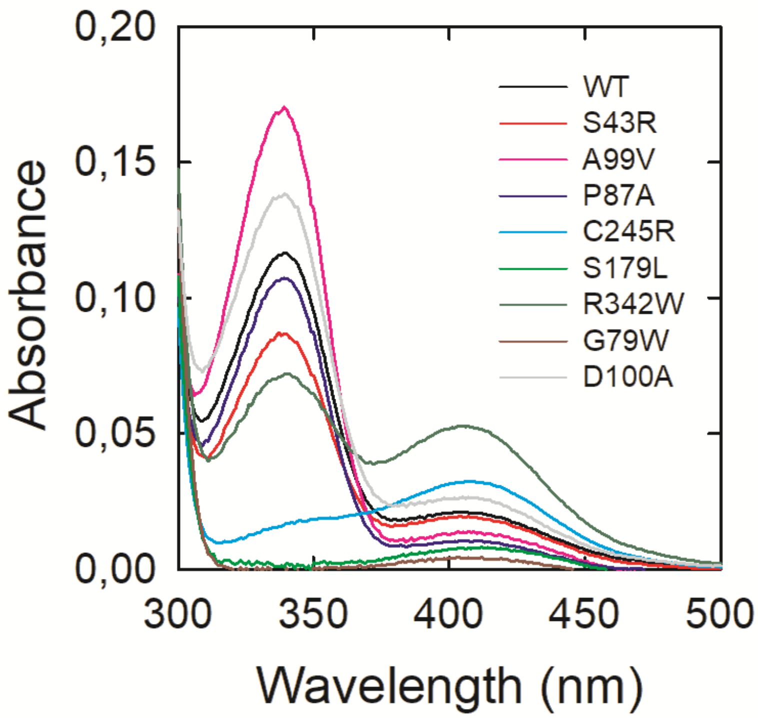

Figure S2: Absorption spectra of 35 µM PSAT variants after purification, in the region of PLP absorption, in a buffer containing 25 mM Tris-HCl, 300 mM NaCl, pH 8.

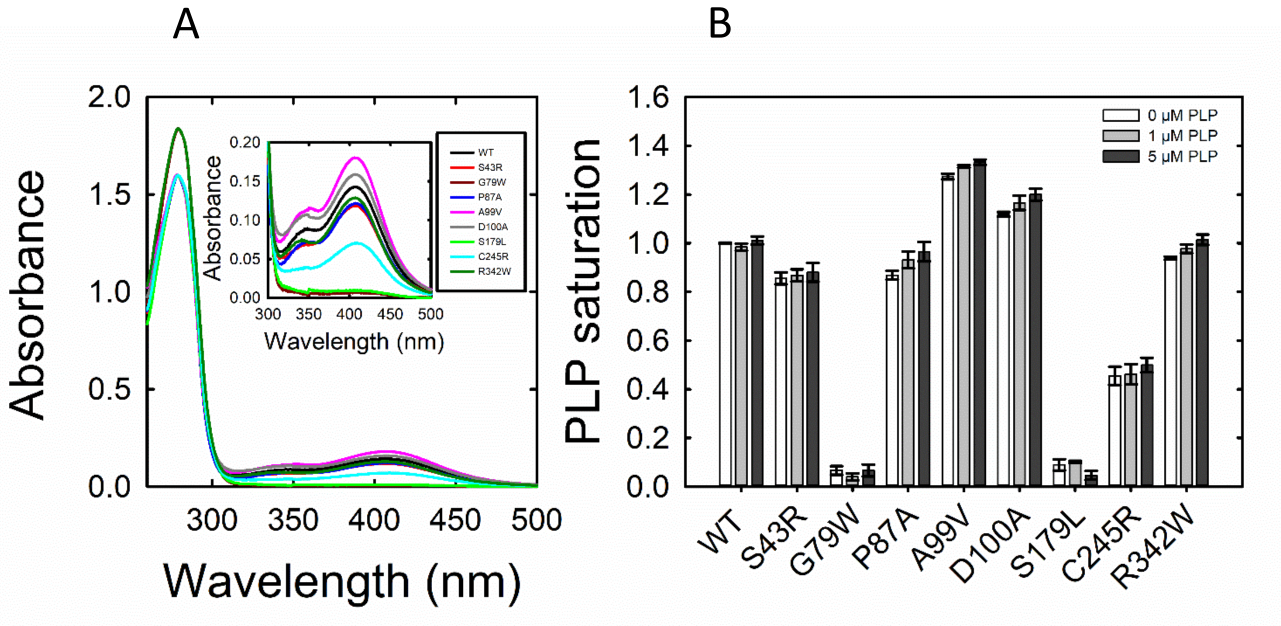

Figure S3: (A) Absorption spectra of 40 µM PSAT-PLP form, in a buffer containing 50 mM MES, 50 mM HEPES, 50 mM bicine, and 100 mM NaCl, pH 5.9. Inset: region of PLP absorption. The spectra were normalized to the same protein concentration, taking into account a different predicted extinction coefficient at 280 nm due to the presence of an additional Trp residue in variants G79W and R342W. (B) PLP saturation - assessed as the ratio of absorption intensity at 408 nm and 280 nm (normalized to that of WT PSAT) for all PSAT variants in the same buffer as in panel A and in the presence of 0, 1 and 5 µM PLP. The proteins were incubated 1 h with PLP before recording their spectra.

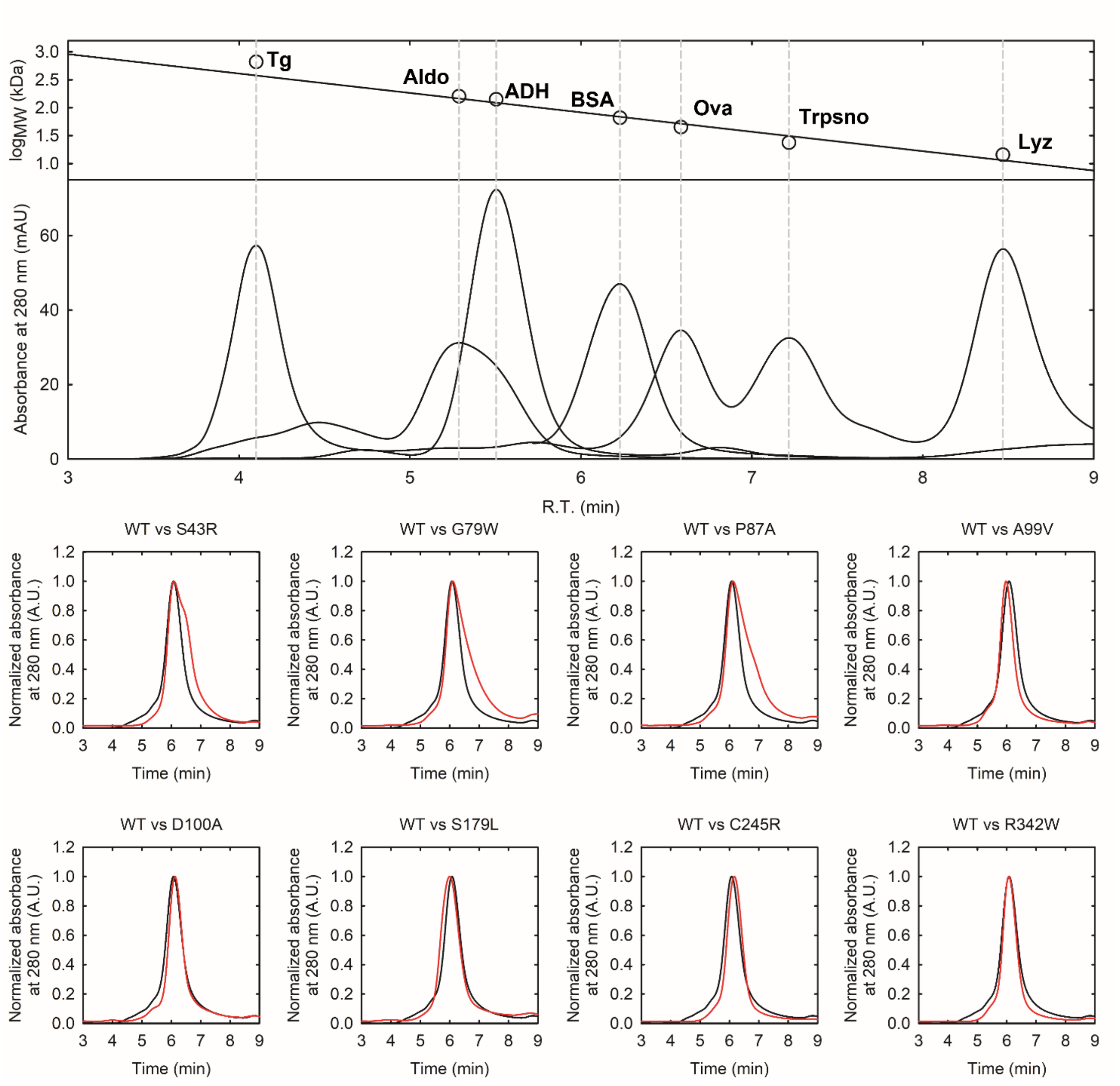

Figure S4: Upper panel: SEC calibration curve obtained using thyroglobulin (Tg), aldolase (aldo), alcohol dehydrogenase (ADH), bovine serum albumin (BSA), ovalbumin (Ova) trypsinogen (Trpsno), lysozyme (Lyz). Lower panel: Overlay of SEC UV traces corresponding to the elution profile for PSAT WT (black) and variants (red). Column: Superdex 200 Increase 5/150 GL column, mobile phase: 50 mM HEPES (pH 7) and 300 mM KCl, flow rate 0.3 mL/min.

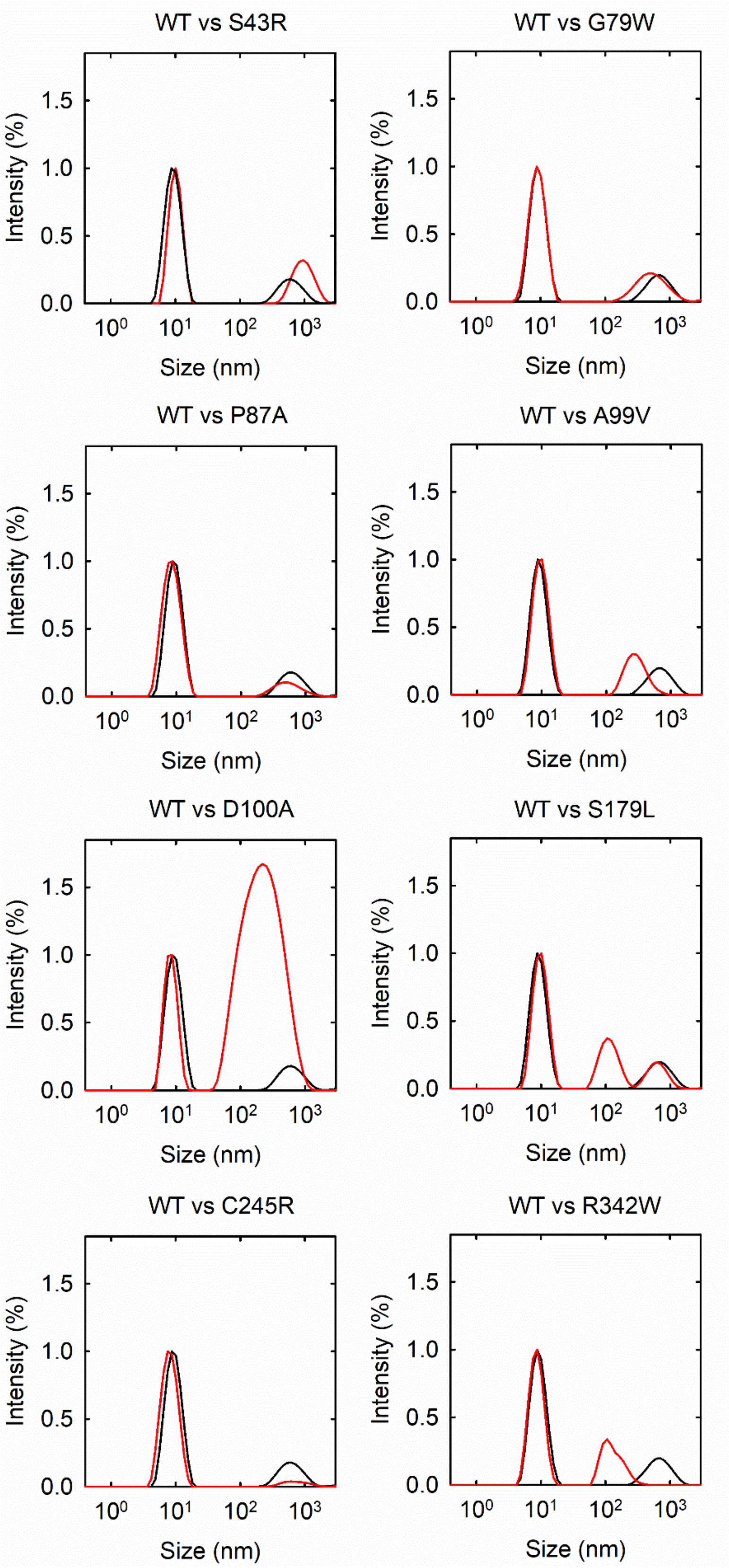

Figure S5a: DLS analysis of 23.3 µM PSAT WT (black) and variants (red) in a buffer containing 50 mM NaH_2PO_4, 300 mM NaCl pH 7. Data were normalized to the peak intensity of the particle corresponding to dimeric PSAT.

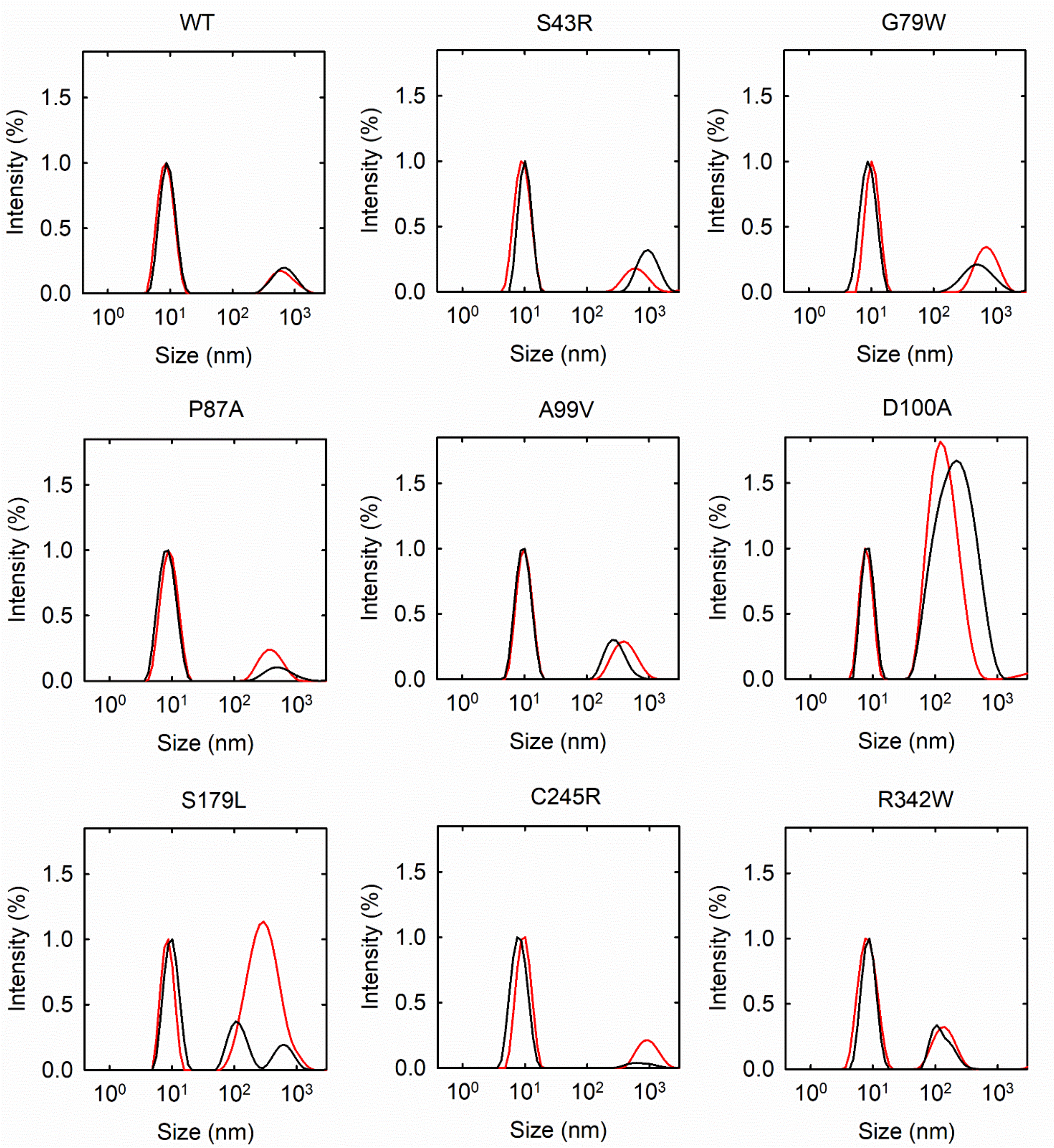

Figure S5b: DLS analysis of 23.3 µM PSAT variants immediately after preparation (black) and after 1 hour (red), in a buffer containing 50 mM NaH_2PO_4, 300 mM NaCl, pH 7. Data were normalized to the peak intensity of the particle corresponding to dimeric PSAT.

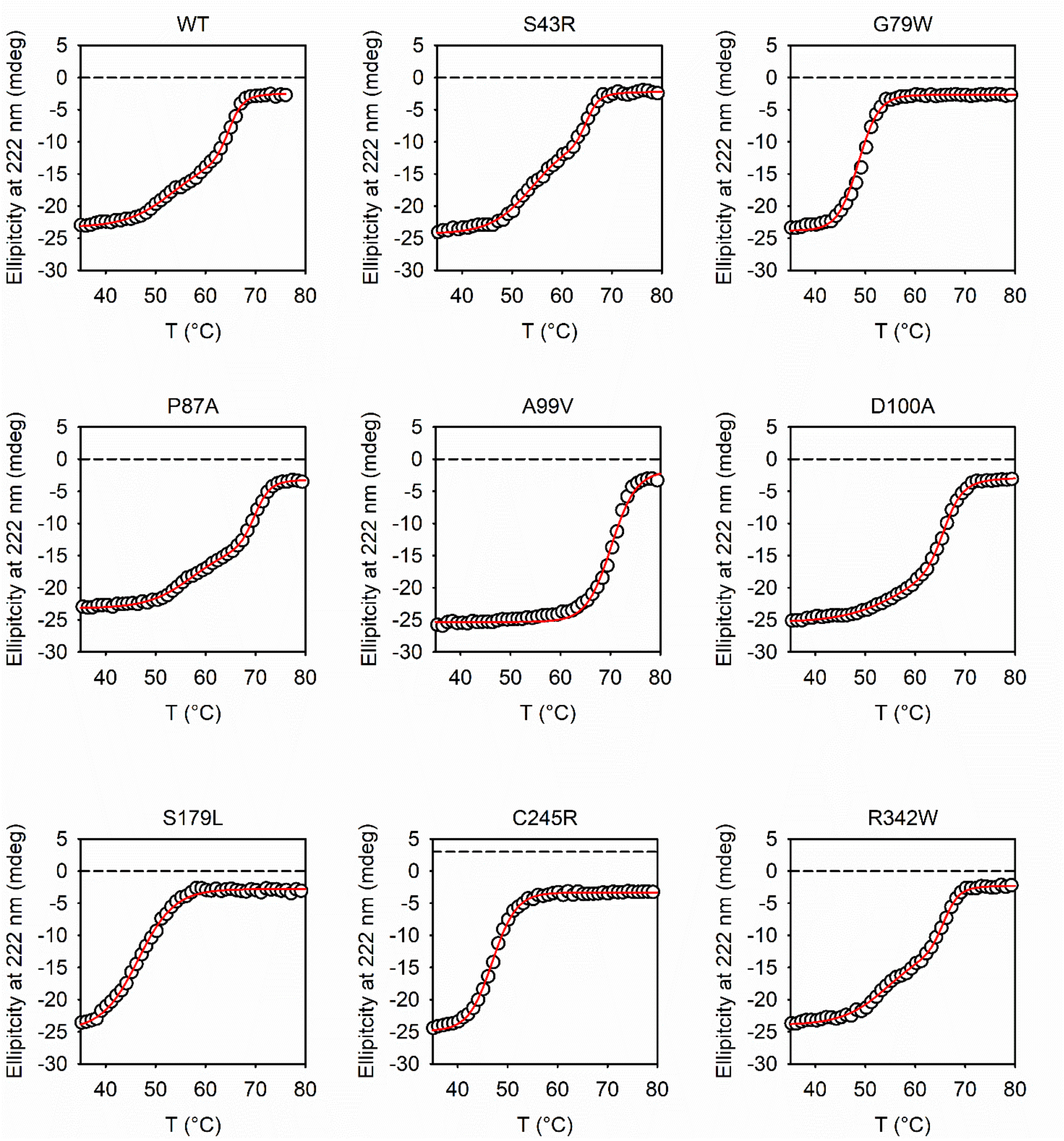

Figure S6: Thermal stability of 5 µM PSAT variants in the 30-80 ˚C range. Data were fitted using the Boltzmann equation for variants showing a monophasic thermal denaturation process (i.e., G79W, S179L, A99V and C245R PSAT) or using a double Boltzmann equation for variants showing a two-step denaturation process (i.e., WT, S43R, P87A, D100A and R342W PSAT).

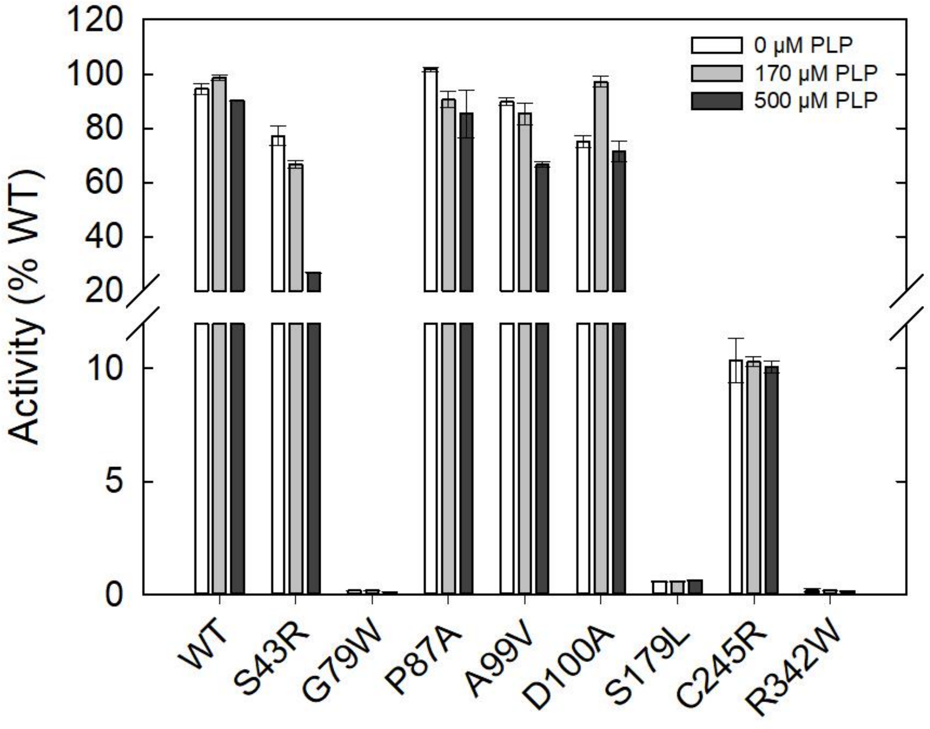

Figure S7: Enzyme activity of 80.8 nM PSAT variants in the presence of 0, 170 and 500 µM PLP. The buffer contained 50 mM HEPES, 100 mM KCl, 100 µM 3-PHP, 130 µM NADH, 20 mM L-Glu, 32 mM NH4Cl and 1.5 mM DTT, pH 7. The enzymes were incubated with PLP for 1 h before the enzyme assays.

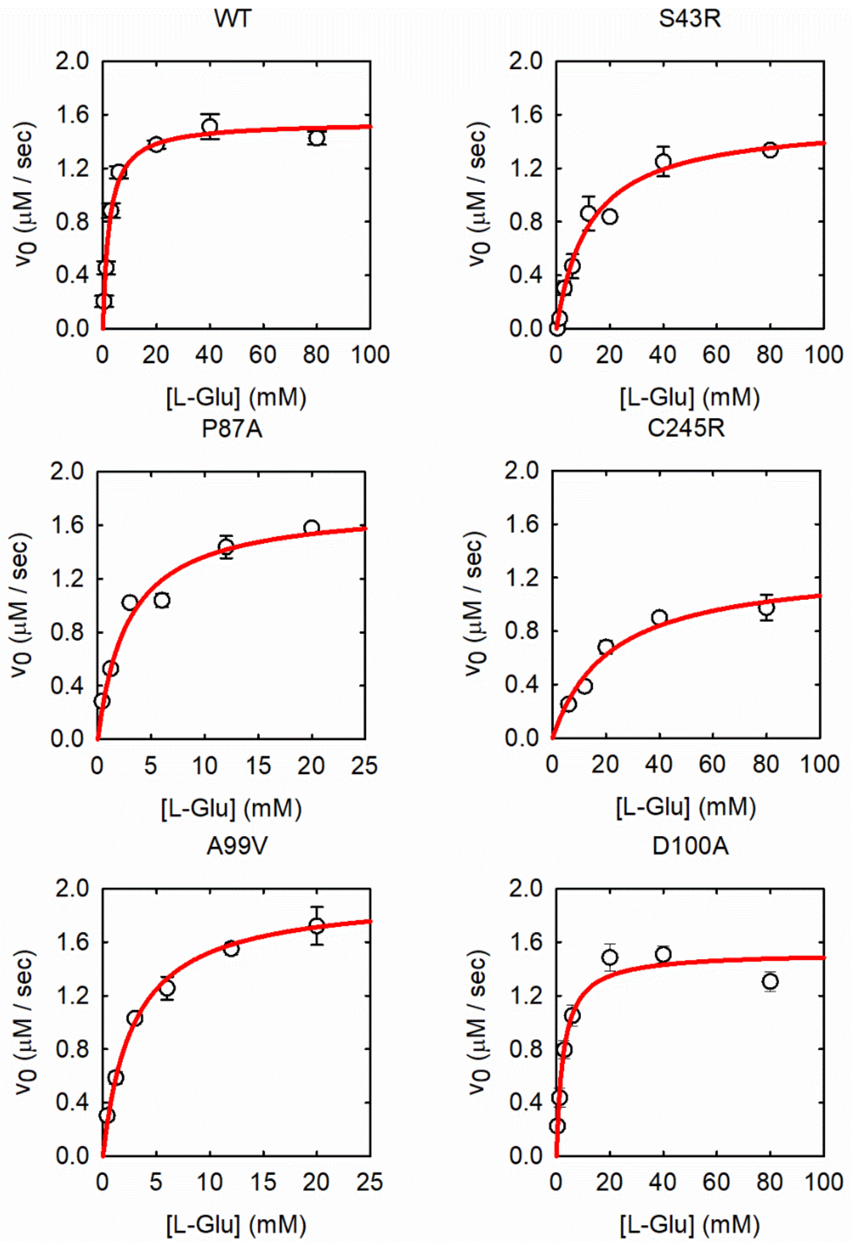

Figure S8: Initial velocity as a function of L-Glu concentration keeping 3-PHP concentration constant at 0.11 mM. The kinetic parameters were determined by fitting the initial rates as a function of L-Glu concentrations to the Michaelis–Menten equation.

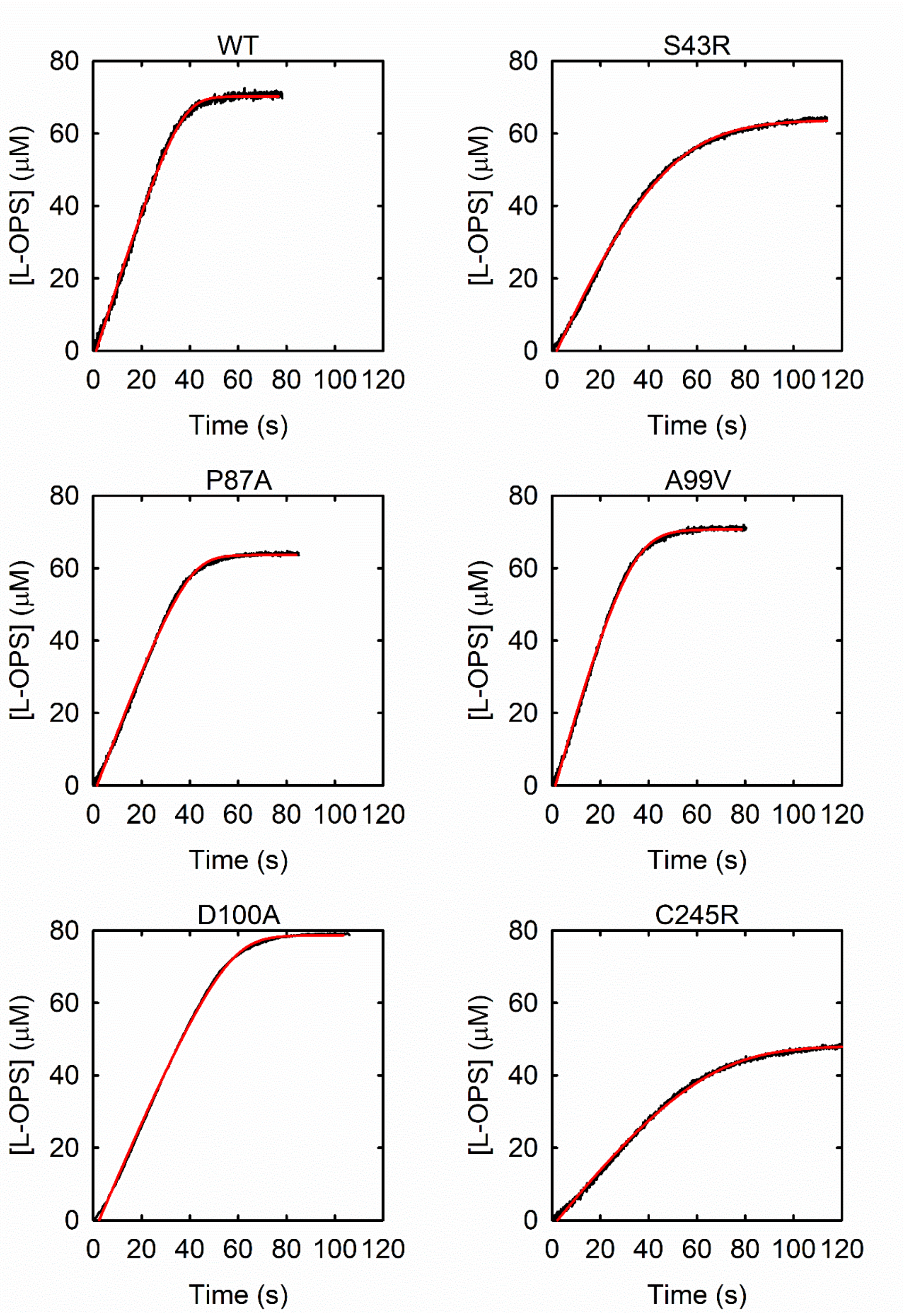

Figure S9: Nonlinear regression of kinetic traces of PSAT using the Schnell – Mendoza equation to obtain the apparent kinetic parameters for 3-PHP. Data were analyzed by the tool PCAT [43] using MATLAB software. The concentration of L-Glu was equal to 5-fold the Km L-Glu value, in order to almost saturate the enzyme variants (10 mM for WT enzyme; 14 mM for A99V and P87A PSAT; 65 mM for S43R PSAT and 108 mM for C245R PSAT).

Table S1: Secondary structure analysis of 5 µM PSAT-PLP CD spectra. DichroWeb software.

	Alpha helix (%)	Beta sheet (%)	Turns (%)	Unordered
**WT**	33.2±0.9	18.0±2.0	18.8±0.6	30.2±0.1
**S43R**	31.9±0.2	19.3±0.5	18.9±0.6	29.9±0.1
**G79W**	33.9±1.0	16.0±1.0	19.4±0.4	31.0±0.2
**P87A**	31.0±3.0	19.0±2.0	19.6±0.3	30.3±0.1
**A99V**	31.0 ±3.0	18.0±2.0	19.9±0.3	30.4±0.1
**D100A**	32.2±0.7	18.7±0.2	18.8±0.8	30.3±0.1
**S179L**	25.9±0.3	23.0±0.3	20.6±0.3	30.4±0.3
**C245R**	28.0±2.0	21.0±2.0	19.9±0.3	30.7±0.1
**R342W**	31.9±0.3	19.0±1.0	19.3±0.9	30.0±0.2

Table S2: Secondary structure analysis of 5 µM PSAT-PMP CD spectra. DichroWeb software.

	Alpha helix (%)	Beta sheet (%)	Turns (%)	Unordered
**WT**	33.0±1.0	18.5±1.0	18.7±0.5	29.7±0.1
**S43R**	30.0±3.0	20.0±2.0	19.6±0.4	29.7±0.2
**G79W**	30.4±0.4	19.0±1.0	19.9±0.2	31.0±0.5
**P87A**	29.0±1.0	21.0±1.3	20.1±0.1	30.2±0.1
**A99V**	33.6±0.3	17.0±1.0	19.3±0.2	29.8±0.6
**D100A**	30.5±0.2	20.2±0.3	19.8±0.2	29.8±0.3
**S179L**	24.5±0.7	23.7±0.9	21.40±0.1	30.6±0.1
**C245R**	28.4±0.9	20.0±1.0	20.70±0.1	30.9±0.1
**R342W**	31.8±2.0	19.5±1.8	19.30±0.1	29.5±0.1
